# COX-2 expression in ovarian cancer: an updated meta-analysis

**DOI:** 10.18632/oncotarget.21538

**Published:** 2017-10-04

**Authors:** Haiming Sun, Xuelong Zhang, Donglin Sun, Xueyuan Jia, Lidan Xu, Yuandong Qiao, Yan Jin

**Affiliations:** ^1^ Laboratory of Medical Genetics, Harbin Medical University, Harbin 150081, China; ^2^ National Human Genome Research Institute, National Institutes of Health, Baltimore, MD 21224, USA

**Keywords:** COX-2, ovarian cancer, meta-analysis, prognosis

## Abstract

The prognostic role of COX-2 expression in ovarian cancer patients has been studied for years, while results remain controversial. Thus we performed a meta-analysis to evaluate the prognostic impact of COX-2 expression on survival of ovarian cancer patients. The databases PubMed, Embase and CNKI were searched. Summary hazard ratio (HR) and 95% confidence intervals (CIs) were calculated to analyze the correlations between COX-2 expression and overall survival (OS), and disease-free survival (DFS). A total of 1,867 patients from 18 studies were enrolled in the final analysis. The results showed that patients with higher COX-2 expression had a poor OS (HR: 1.48; 95% CI: 1.19-1.85) and DFS (HR: 1.81, 95% CI: 1.28-2.55). Subgroup analysis showed that there had significant associations between COX-2 expression and survival rate in most of the subgroups. Furthermore, there were significant associations between COX-2 expression and several clinical parameters such as FIGO stage, histological type and age. These results showed the patients with higher COX-2 expression had a significantly poorer survival rate, COX-2 expression had the potential to be a prognostic marker of ovarian cancer.

## INTRODUCTION

Ovarian cancer ranks the fifth as a cause of neoplastic death among women, there would be more than 22,000 new cases and over 14,000 deaths in the United States in 2017 [1]. Because early-stage tumors are typically asymptomatic, most patients have advanced stage at the time of diagnosis, resulting in a poorer long-time survival [2, 3]. The overall 5-year survival rate of ovarian cancer is just approximately 30% [4]. Given the poor survival rates of ovarian cancer, it is necessary to identify prognostic biomarkers for effectively evaluating the outcomes of the patients.

The cyclooxygenase-2 (COX-2) is called the prostaglandin-endoperoxide synthase-2 (PTGS-2) too, it involved in the processes of inflammatory and oncogenic [5, 6]. Furthermore, COX-2 dependent prostaglandin release can inhibit antigen presentation and immune activation during carcinogenesis [7]. COX-2 overexpression was found in most solid tumors, such as breast, colorectal, lung, pancreatic, liver, as well as ovarian cancer [8–11]. And some studies have reported the prognostic role of the expression of COX-2 in ovarian cancer, while the results are varying and sometimes conflicting. In order to clarify the issues, we collected the eligible articles and performed a meta-analysis to assess the prognostic impact of COX-2 expression in the patients with ovarian cancer.

## RESULTS

### Literature searching and study characteristics

**Figure 1:**
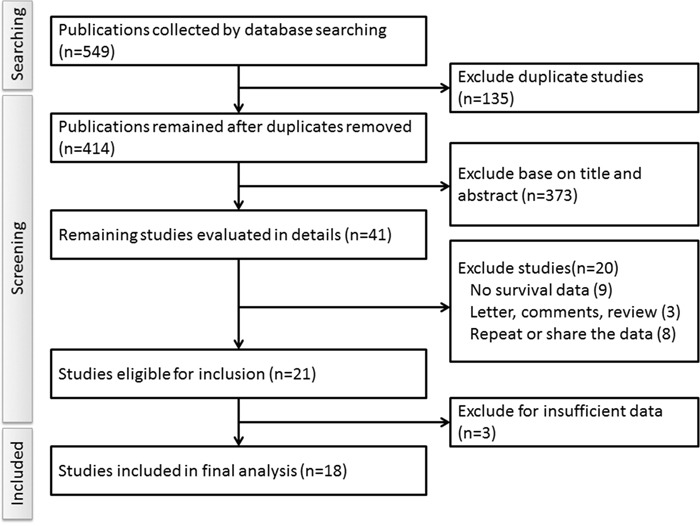
Flow chart of the literature search

By the keywords searching, a total of 547 publications were found. Based upon the selection criteria, a total of 18 publications were identified and included in the final analysis [12–29]. The details of the procedures of literature screening were shown in Figure 1. The total number of ovarian cancer patients included in the meta-analysis was 1,867, the mean sample size was 103 (ranges from 32 to 442). Among them, 18 studies reported the overall survival (OS) and 4 for disease-free survival (DFS). In the study of Ferrandina [28], they presented the data in two subgroups, so we treated the study as two datasets. We extracted hazard ratio (HR) and a 95% confidence interval (CI) from the Kaplan-Meier curves in 10 articles. The main characteristics of the included studies were listed in Table 1.

**Table 1:** Main characteristics of the studies included in this meta-analysis

Study ID	Country	Sample size	Median or mean age/range(year)	FIGO stage	Histological subtype	Follow-up time (months)	COX-2 detection method	High expression cut-off level	Number of high expression patients	Outcome (OS/DFS)	Study quality	Source of HR
Ali-Fehmi (2011)	USA	126	57.6	I-IV	serous	54 (1-235)	IHC	Staining intensity ≥2 and stained cells >10% or staining intensity ≥1 and stained cells >50% staining intensity ≥1 and stained cells >50%	96	OS	9	R
Denkert (2002)	Germany	86	NA	I-IV	Serous, undifferentiated, nonserous	32.5 (0.3-121.7)	IHC	Diffuse staining or a focal expression in several clusters of cells	36	OS	5	R
Seo (2004)	South Korea	64	51	I-IV	serous, metrioid, mucinous	56 (6-68)	IHC	>5% of cells were positively stained for mucinous or >30% for serous and endometrioid	64	OS	8	R
Raspollini (2004)	Italy	78	58	III	serous	47 (3-204)	IHC	Positive staining >10% of the total tumor area of intensity of staining scored ≥2	54	OS/DFS	8	R
Ferrero (2011)	Italy	113	62	II-IV	Serous, mucinous, endometrioid, undifferentiated	NA	IHC	Staining intensity ≥2 and >10% stained cells or staining intensity ≥1 and >50% stained cells	45	OS	7	R
Surowiak (2006)	Poland	43	NA	III	Serous, endometrioid	NA	IHC	Stained in all tumors cells or in numerous cell clumps	19	OS/DFS	6	E
Wang (2011)	China	147	43.15	I-IV	Serous, mucinous and others	NA	IHC	Staining intensity ≥2 or percentage of stained cells ≥ 30%	109	OS	5	R
Athanassiadou (2008)	Greece	100	62	I-IV	Serous, mucinous, endometrioid, undifferentiated	67.12	IHC	Staining reaction >10%	56	OS	6	E
Erkinheimo (2004)	Finland	442	57	I-IV	Serous	5.2 years (0.4-36.1)	IHC	Staining in >10% cancer cells	310	OS	9	E
Khalifeh (2004)	USA	96	62	III, IV	Serous	35.3	IHC	Intensity 2 or 3 and >10% and /or intensity 1,2 or 3 and >50%	65	OS	7	E
Steffensen (2007)	Denmark	160	54.5	II-IV	Serous, mucinous, endometrioid, undifferentiated and others	more than 10 years	IHC	>10% of the total tumor area showing moderate or strong immunostaining	32	OS	7	R
Magnowska (2014)	Poland	65	NA	NA	Serous and others	37.2 (24-74)	IHC	Immunoreactivity Score >6	33	OS/DFS	5	R
Taskin (2012)	Turkey	32	58.63	II-III	Serous	33.7 (8-124)	IHC	The multiplied staining intensity and stained cell percent >3	15	OS	7	E
Lou (2004)	China	70	54	I-IV	Serous, mucinous, endometrioid, undifferentiated and others	31 (5-71)	IHC	Staining in >10% cancer cells	42	OS	6	E
Lee (2006)	USA	54	51	I-IV	Serous, mucinous, endometrioid and clear cell	67 (3-119)	IHC	Staining intensity of 2 or 3 and >10% stained cells or an intensity 1 and >50% stained cells	42	OS	7	E
Fujimoto (2006)	Japan	60	NA	I-III	Serous, mucinous, endometrioid	up to 24 months	ELISA	>14 ng/mg protein	30	OS	6	E
Ferrandina (2002)	Italy	87	57	III, IV	Serous, mucinous, endometrioid, undifferentiated and others	25 (4-147)	IHC	>10% of the total tumor area or intensity of staining ≥2	39	OS/DFS	6	E
Ozuysal (2009)	Turkey	44	54.2	I-IV	Serous	40	IHC	Staining intensity ≥ 2 and percentage >10% or staining intensity ≥ 1 and percentage >50%	17	OS	7	E

IHC: immunohistochemistry; NA: not available; OS: overall survival; DFS: disease-free survival; EILSA: enzyme-linked immunosorbent assay; R: reported in the articles; E: estimated from Kaplan-Meier plots.

### COX-2 expression and OS

**Figure 2:**
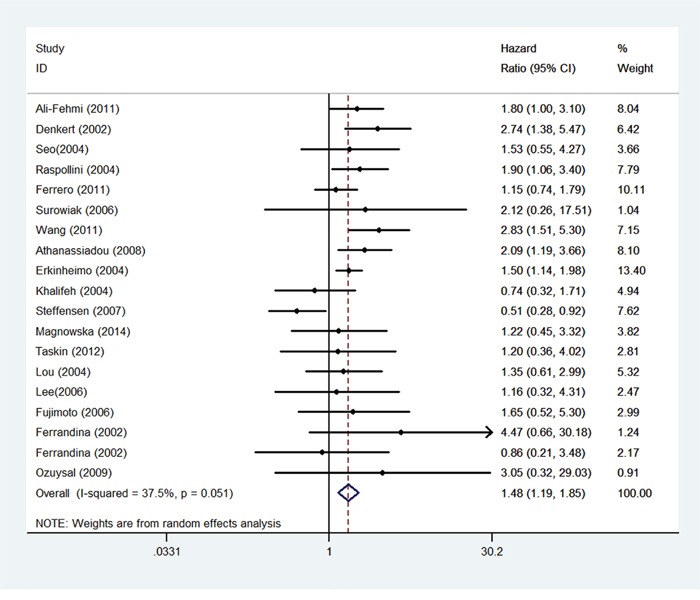
Forest plots of hazard ratios (HRs) for the association between COX-2 expression and overall survival (OS) in ovarian cancer patients The 95% confidence intervals (CI) for individual studies are represented by a horizontal line and by a diamond for pooled effect. CI denotes confidence interval.

Firstly, we analyzed the impact of COX-2 expression levels on OS in 18 studies. The random-effects model was used to estimate the pooled HRs and the respective 95% CI. The results showed that the patients with higher COX-2 expression had a significantly increased risk of death than the patients with lower COX-2 expression (Figure 2, HR: 1.48; 95% CI: 1.19-1.85). Then, we performed subgroup analysis according to study quality, sample size, follow-up time, histology subtypes, regions of the study and calculation methods of HR. Based on the region of study, we found patients with higher COX-2 expression had a poor prognosis in European and Asian studies. When subgroups were stratified by the statistical analysis methodology, the results demonstrated that the survival rates had an obvious distinction between lower and higher expression groups both by univariable analysis and multivariable analysis. When restricted to the histology types of the cancer, we found that patients with higher COX-2 expression had a poor survival rate not only in serous ovarian but also in the total types of ovarian cancer. No matter the length of the follow-up time, the patients with a higher COX-2 expression had a lower overall survival rates. But the results did not have statistical significant in two subgroups. All of the details were listed in Table 2.

**Table 2:** Main results of the meta-analysis

Categories	Number of datasets	HR	95%CI	*P*	Degree of heterogeneity (I^2^ statistics; %)
OS	19	1.48	1.19-1.85	<0.001	37.5
Study quality					
Score ≥ 7	10	1.24	0.93-1.65	0.136	44.3
< 7	9	2.04	1.53-2.71	<0.001	0
Sample size					
≥ 100	6	1.44	0.98-2.13	0.065	74.3
< 100	13	1.57	1.19-2.07	0.001	0
Duration of follow-up (Months)					
> 36	9	1.42	1.04-1.95	0.029	48.7
≤ 36	10	1.56	1.11-2.20	0.01	31.2
Histology types					
All	13	1.49	1.06-2.09	0.021	50.9
Serous	6	1.52	1.22-1.88	<0.001	0
Region					
European	12	1.44	1.07-1.95	0.016	48.3
North American	3	1.25	0.69-2.26	0.456	33.8
Asian	4	1.95	1.29-2.95	0.002	0
Analysis type					
Multivariate	8	1.52	1.01-2.28	0.044	68.2
Univariate	11	1.49	1.21-1.84	<0.001	0

HR: hazard ratio; CI: confidence interval; OS: overall survival.

### COX-2 expression and DFS

**Figure 3:**
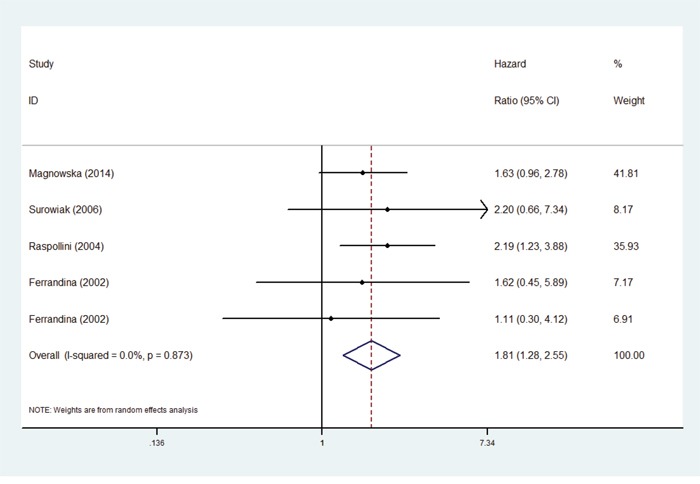
Forest plots of hazard ratios (HRs) for the association between COX-2 expression and disease-free survival (DFS) in ovarian cancer patients The 95% confidence intervals (CI) for individual studies are represented by a horizontal line and by a diamond for pooled effect. CI denotes confidence interval.

In the enrolled studies, there were four studies reported the associations between COX-2 expression and DFS [15, 17, 23, 28], in which, the study of Ferrandina included two datasets. The results indicated that the patients with higher COX-2 expression had a poor disease-free survival (Figure 3, HR: 1.81, 95% CI: 1.28-2.55).

### Sensitivity analysis and publication bias

**Figure 4:**
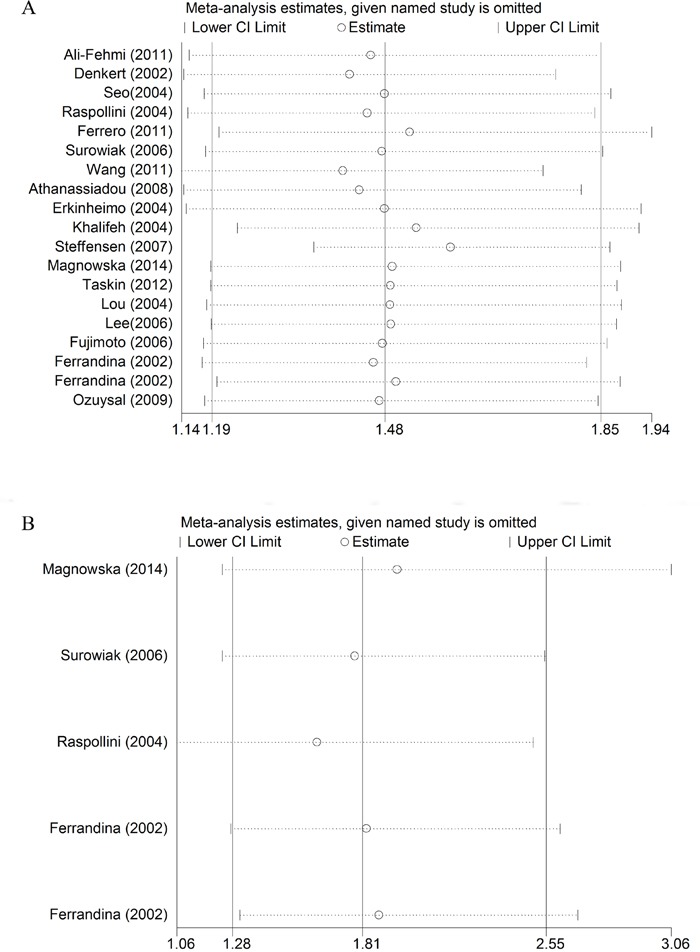
Sensitive analysis of the pooled hazard ratio for OS **(A)** and DFS **(B)**. Meta-analysis random effects estimates were used. Results were computed by omitting each study (on the left) in turn. The two ends of every broken line represented the 95% confidence interval.

**Figure 5:**
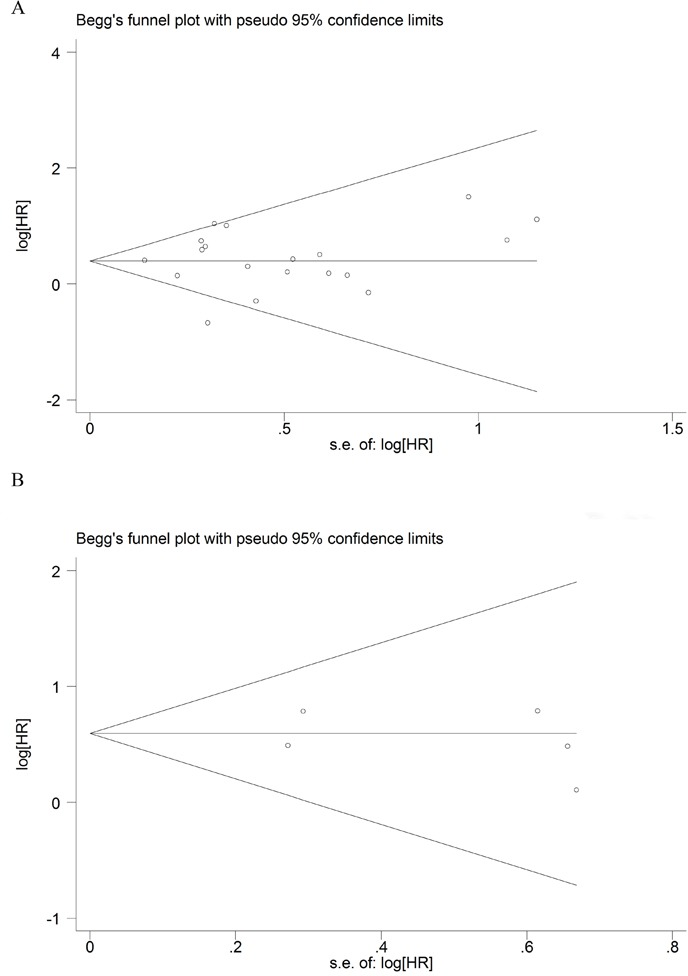
Funnel plot for the assessment of publication bias of the included literature for OS **(A)** and DFS **(B)**.

In order to evaluate the influence of the single study to the final results, sensitivity analysis was carried out by deleted single study each time. As shown in Figure 4, there was no significant change on the results by removing any of the studies, which mean that our results were reliable. Begg's funnel plots were constructed to evaluate the publication bias; we did not find the sign of publication bias by the shapes of the plots (Figure 5). The results of Egger's test showed there were no publication bias, too (*P* = 0.892 for OS; *P* = 0.599 for DFS).

### Cox-2 expression and clinical parameters

We analyzed the associations between COX-2 expression and different clinical parameters such as age, FIGO stage, histological type, et al. Pooled ORs showed that COX-2 expression correlated with FIGO stage (I/II vs. III/IV), histological type (serous vs. others) and age (young vs. old), while no significant correlations were found between COX-2 expression and tumor grade, lymph node transmission and menopausal status. Then we carried out the sensitivity analysis to evaluate the influence of the single study to the final results, the results did not change in the analysis of FIGO stage, tumor grade, lymph node transmission and menopausal status, while the results changed in the analysis of age and histological type when we deleted one single study (data not show). And there was no publication bias in all of these analysis. The details were showed in Table 3.

**Table 3:** Association of COX-2 expression with clinical features in ovarian cancer

Clinical parameter	Number of studies	OR	95%CI	P^*^
Age (young vs. old)	7	1.37	1-1.81	0.126
FIGO stage (I/II vs. III/IV)	9	2.41	1.28-4.53	0.128
Histological type (serous vs. others)	7	0.69	0.48-0.98	0.119
Tumor grade (Low 1/2 vs. high 3)	11	1.2	0.76-1.89	0.858
Lymph node transmission (positive vs. negative)	3	2.36	0.78-7.11	0.831
Menopausal status (pre vs. post)	2	1.2	0.45-3.2	-

Abbreviations: OR: odds ratio; CI: confidence interval; ^*^P value for publication bias.

## DISCUSSION

COX-2 is the rate-limiting enzyme involved in the conversion of arachidonic acid to various prostaglandins. Studies showed that COX-2 overexpressed in inflammatory and many malignancies [30–32]. The prognostic impact of COX-2 expression had been reported in different tumors [33–36]. Its role in ovarian cancer patients had been reported in several studies, while the results were inconsistent. These conflicting might be affected by the relatively limited sample size included in individual studies. Meta-analysis is an efficient way to combine small studies and get reliable results.

A previous meta-analysis reported the prognostic impact of COX-2 on ovarian cancer mortality by Lee et al [37]. But there are some issues we should concern in their article. First, they mentioned that there were 17 studies included in their analysis, but there were no data for the studies of Sakamoto [38] and Menczer [39]. Second, there were some mistakes in the data extraction for the studies of Ferrero [16] and Ferrandina [28]. Lee extracted the results of univariate analysis while not multivariate from Ferrero. In the study of Ferrandina, there were two Kaplan-Meier plots for different subgroups, while Lee just extracted one of them to represent the total patients. Furthermore, there are some new data available, so, the purpose of this study was to update the meta-analysis and get a more accruable result.

The present meta-analysis, which included 1,867 ovarian patients from 18 studies, indicated that higher COX-2 expression was a signal of poor prognosis for ovarian cancer patients. Our subgroup analysis by follow-up times revealed that the higher expression of COX-2 was strongly associated with poor prognoses regardless the length of the follow-up time. Similarly, we found significant associations between COX-2 overexpression and survival rate in most subgroup analysis. Furthermore, we found there were significant correlations between COX-2 expression and FIGO stage, histological type and the age of patients. Based on these results, COX-2 could be a valuable prognostic biomarker and a therapy target for ovarian cancer patients in clinical trials.

We did not totally understand the exact mechanism by which COX-2 overexpression causes poor prognosis in ovarian cancer patients. COX-2 expression could be induced by various factors, such as mitogens, cytokines, and prostaglandins [40]. It had been shown that COX-2 plays an essential role during tumorigenesis [7, 41]. The overexpression of COX-2 could cause the growth, invasion, migration of ovarian tumor cell, and chemoresistance in ovarian cancers patients [11, 19, 42]. All of these could reduce the survival rate of the patients. More studies are required to investigate the specific molecular mechanism of COX-2 overexpression reducing the survival rate of ovarian cancer patients.

Heterogeneity and publication bias are two important factors to evaluate a meta-analysis. In this study, heterogeneity test revealed there was no obvious heterogeneity in the main analysis and most of the subgroup analysis. The heterogeneity appeared in several subgroups analysis (sample size ≥100 and multivariate analysis). It may partly come from various antibodies, different staining scores or different cut-off values for the higher COX-2 expression level among different studies. So selection of appropriate staining scores and cut-off values would help to improve the reliability of the results and reduce the heterogeneity among different studies. As for publication bias, no proof of publication bias was found. These findings suggested that the results of the meta-analysis were reliable. However, the present study still had some limitations. First, this study is based on literature searching; it is possible that there are some studies were not included in the analysis, even though public bias was not found in this study. Second, most of the enrolled studies just had limited patients, the sample size was small. Third, the number of enrolled studies is relatively small for Asian and North American in the subgroup analysis by study region.

In conclusion, the patients with higher COX-2 expression had a significant poorer OS and DFS. COX-2 expression could be a prognostic marker to help to define high risk patients and find novel therapeutic target for ovarian cancer. But more studies, especially the studies with large number of patients, were needed to clarify the relationships between COX-2 expression and survival rate of ovarian cancer patients.

## MATERIALS AND METHODS

### Literature searching and study selection

PubMed, Embase, and CNKI were used to retrieve potentially eligible studies published by December 2016. The keywords for the search in these databases included: “ovarian cancer” or “ovarian carcinoma” or “ovarian tumor” or “ovarian tumour” or “ovarian neoplasm” or “ovarian malignancy”, “COX-2” or “Cyclooxygenase-2”, and “prognosis” or “survival” or “outcome” or “mortality”. In addition, we also conducted the manual searches of references in all eligible studies to identify potential missing publications. There were no any restrictions on the searches.

Studies would not be considered unless they met the following criteria: provided survival data in ovarian cancer patients stratified by COX-2 expression and sufficient data to calculate an estimate of hazard ratio (HR) and a 95% confidence interval (CI); all selected ovarian cancer patients were pathologically confirmed and the protein expression of COX-2 was measured in the ovarian cancer specimen. All articles were reviewed to avoid the duplicate data. The most recent or most complete publication was enrolled if there were overlaps between studies.

### Data extraction and quality assessment

Two investigators conducted the literature review and extracted data by using a standardized data extraction form, separately. For each article, the following information was extracted: first authors and the year of publication, sample size, country, FIGO stage, histological subtype, follow-up time, COX-2 detection methods, cut-off level of the higher expression, number of high expression patients, HR and 95% CI and methods of HR estimation. For the studies which provided both the univariate and multivariate analysis, HR and 95% CI of multivariate analysis were extracted because they could avoid the interference of confounding factors. If the results of survival analysis were not reported by the authors, we extracted the survival data from the Kaplan-Meier curves and calculated HR and 95% CI by the methods of Jayne et al [43]. For the studies which provided the relationships between COX-2 expression and clinical parameters, we extracted the related data. We used the New-castle-Ottawa Scale (NOS) to evaluate the quality of each study [44]. Studies were assigned to high quality group if they had the score of 7 or higher. The extracted data were crosschecked and disagreements were resolved by discussion.

### Statistical analysis

The two outcomes endpoints were overall survival (OS) and disease-free survival (DFS). Pooled HRs with 95% CIs were calculated based on random-effects model due to the possible substantial heterogeneity among studies [45]. In order to evaluate the relationships between COX-2 expression and clinical parameters, pooled ORs and 95% CIs were calculated based on random-effects model too. Heterogeneity was examined by I^2^ statistic, I^2^ ≥ 50% indicated the presence of significant heterogeneity [46]. We further investigated potential heterogeneity by subgroup analyses stratified by study quality, sample size, follow-up time, histology subtypes, region of the study, and calculation methods of HR. Sensitivity analysis was conducted to evaluate the stability of the results. Begger's funnel plots and Egger's test were used to find the potential publication bias [47, 48]. All statistical analyses were performed by Stata statistical software (version 11.0; Stata Corporation, College Station, TX, USA).
